# Transcriptomic meta‐analysis of disuse muscle atrophy vs. resistance exercise‐induced hypertrophy in young and older humans

**DOI:** 10.1002/jcsm.12706

**Published:** 2021-05-05

**Authors:** Colleen S. Deane, Craig R.G. Willis, Bethan E. Phillips, Philip J. Atherton, Lorna W. Harries, Ryan M. Ames, Nathaniel J. Szewczyk, Timothy Etheridge

**Affiliations:** ^1^ Department of Sport and Health Sciences, College of Life and Environmental Sciences University of Exeter, St. Luke's Campus Exeter UK; ^2^ Living Systems Institute University of Exeter Exeter UK; ^3^ MRC‐ARUK Centre for Musculoskeletal Ageing Research and National Institute of Health Research, Biomedical Research Centre, Division of Medical Sciences and Graduate Entry Medicine, Royal Derby Hospital Centre, School of Medicine University of Nottingham Derby UK; ^4^ RNA‐Mediated Mechanisms of Disease Group, Institute of Biomedical and Clinical Sciences University of Exeter Medical School, University of Exeter Exeter UK; ^5^ Ohio Musculoskeletal and Neurological Institute & Department of Biomedical Sciences Ohio University Athens OH USA

**Keywords:** Skeletal muscle disuse, Resistance exercise training, Ageing, Transcriptomic meta‐analysis, Gene‐level analysis, Network analysis

## Abstract

**Background:**

Skeletal muscle atrophy manifests across numerous diseases; however, the extent of similarities/differences in causal mechanisms between atrophying conditions in unclear. Ageing and disuse represent two of the most prevalent and costly atrophic conditions, with resistance exercise training (RET) being the most effective lifestyle countermeasure. We employed gene‐level and network‐level meta‐analyses to contrast transcriptomic signatures of disuse and RET, plus young and older RET to establish a consensus on the molecular features of, and therapeutic targets against, muscle atrophy in conditions of high socio‐economic relevance.

**Methods:**

Integrated gene‐level and network‐level meta‐analysis was performed on publicly available microarray data sets generated from young (18–35 years) *m. vastus lateralis* muscle subjected to disuse (unilateral limb immobilization or bed rest) lasting ≥7 days or RET lasting ≥3 weeks, and resistance‐trained older (≥60 years) muscle.

**Results:**

Disuse and RET displayed predominantly separate transcriptional responses, and transcripts altered across conditions were mostly unidirectional. However, disuse and RET induced directly inverted expression profiles for mitochondrial function and translation regulation genes, with *COX4I1*, *ENDOG*, *GOT2*, *MRPL12*, and *NDUFV2*, the central hub components of altered mitochondrial networks, and *ZMYND11*, a hub gene of altered translation regulation. A substantial number of genes (*n* = 140) up‐regulated post‐RET in younger muscle were not similarly up‐regulated in older muscle, with young muscle displaying a more pronounced extracellular matrix (ECM) and immune/inflammatory gene expression response. Both young and older muscle exhibited similar RET‐induced ubiquitination/RNA processing gene signatures with associated *PWP1*, *PSMB1*, and *RAF1* hub genes.

**Conclusions:**

Despite limited opposing gene profiles, transcriptional signatures of disuse are not simply the converse of RET. Thus, the mechanisms of unloading cannot be derived from studying muscle loading alone and provides a molecular basis for understanding why RET fails to target all transcriptional features of disuse. Loss of RET‐induced ECM mechanotransduction and inflammatory profiles might also contribute to suboptimal ageing muscle adaptations to RET. Disuse and age‐dependent molecular candidates further establish a framework for understanding and treating disuse/ageing atrophy.

## Background

As the largest tissue in the body, the functions of skeletal muscle extend beyond locomotion and structural support,^1^ providing storage for glucose^2^ and lipids^3^ used for energy production and providing the largest amino acid reservoir for systemic release in times of organismal need.^4^ Thus, muscle atrophy associates with increased risk of frailty‐related falls,^5^, ^6^ increased incidence of metabolic disease,^7^ and ultimately, death.^8^ Indeed, muscle atrophy is a prominent feature of several of the World's key health challenges including ageing, cardiovascular disease, obesity, diabetes, and cancer.^7^, ^9^ As a result, the most recently available estimates of annual cost for age‐related atrophy (sarcopenia) alone is $18.5 billion (USA, 2000)^10^ and £2.5 billion (UK, 2019).^11^ Despite the high socio‐economic relevance of maintaining healthy muscle mass, the mechanisms regulating muscle atrophy and, conversely, hypertrophy are incompletely understood. There is, therefore, a need to establish robust molecular features of atrophy and hypertrophy in order to efficiently promote targeted therapeutics with efficacy across atrophying conditions.

Two of the most prominent lifestyle‐associated atrophic factors include muscle disuse and ageing. For example, muscle atrophy has been reproducibly detected during periods of disuse lasting ≥7 days, as occurs with acute hospitalization, injury, illness, and inactivity.^12^, ^13^, ^14^ Thus, despite being relatively understudied, the health consequences of disuse places inactivity as one of the top five causes of death globally.^15^ Slower atrophy also occurs as an inevitable consequence of the ageing process, with muscle mass declining at rates of ~0.5–1.2% per year from the age of 50.^16^ Disuse and ageing, therefore, represent phenotypically and aetiologically interlinked atrophic stimuli, with repeated periods of disuse likely serving a central causative role in age‐related muscle decline.^13^

Resistance exercise training (RET) remains the most effective non‐pharmacological intervention to mitigate and recover from disuse^17^, ^18^ and to attenuate progression of age‐related muscle atrophy.^19^ Teleologically, the central molecular drivers of RET‐induced hypertrophy might be the direct inverse of those governing disuse‐atrophy; indeed, mitochondrial and extracellular matrix (ECM) gene signatures display opposing expression during RET vs. disuse.^20^, ^21^, ^22^ Similarly, key regulators of the well‐established blunted ageing hypertrophic response to RET^23^ might exactly oppose ‘normal’ RET responses in younger people and/or cluster to entirely distinct molecular pathways. Nonetheless, few studies have directly examined the muscle transcriptome of RET in comparison with either disuse or ageing RET responses,^24^, ^25^, ^26^ precluding a consensus on robust molecular features. Contributing to this modest progress is certainly the significant technical and financial difficulty associated with performing sufficiently controlled human disuse/ageing/RET clinical trials from which cause‐and‐effect can be inferred.^27^ However, the emergence of OMIC technologies has advanced the mechanistic insight possible from human randomized controlled trials. Indeed, recent meta‐analysis of differential gene expression found a ‘mRNA metabolism’ signature inverted between disuse and RET, as well as distinct profiles characterizing RET (increased ECM remodelling) and disuse (reduced mitochondrial pathways and increased ubiquitination) in young‐middle aged adults.^28^ Exploiting big data can thus begin to identify robust transcriptional patterns characterizing atrophic and hypertrophic adaptations.

Disuse and RET transcriptomic studies are also often characterized by differing methodologies (e.g. baseline volunteer characteristics and experimental protocol employed), variable control of key confounders (e.g. diet and activity status), and an individual lack of statistical power, hindering the discovery of robust biosignatures. As such, transcriptomic meta‐analysis offers a judicious strategy to overcome these limitations and aid biomarker discovery within disuse/(ageing) RET muscle adaptation. Indeed, the integration of multiple related transcriptomic data sets into a single analysis has improved the power to confirm/detect novel biosignatures in other societally important pathophysiological scenarios such as cancer and diabetes^29^, ^30^ and recently, to identify global transcriptional responses to exercise and inactivity^28^ and genes that correlate with exercise‐induced changes in muscle mass.^26^ As a statistical approach, transcriptomic meta‐analysis has been routinely applied to identify robust gene‐level expression changes. Nevertheless, the utility of meta‐analysis can be further extended to the network‐level, where molecular complexity is accounted for by modelling gene–gene interactions in the form of a co‐expression network, allowing the identification of concordant patterns of gene co‐regulation associated with physiological phenotypes.^31^ Network‐level meta‐analysis therefore offers a more holistic, biologically‐driven view of conserved molecular mechanisms regulating physiological phenomena, while gene‐level meta‐analysis remains valuable in the selection process of candidate genes.^32^ Thus, combining gene‐level and network‐level meta‐analyses should present a powerful framework for identifying the most biologically relevant and robust candidate targets.^32^ To this end, we applied an integrated transcriptomic meta‐analysis framework to publicly available data with the aim of identifying robust molecular pathways and gene candidates driving: (i) divergent responses to RET vs. disuse and (ii) age‐related responses to RET.

## Methods

### Transcriptomic data mining

Relevant transcriptomic data sets were sought via data mining of the Gene Expression Omnibus and ArrayExpress public repositories: up to and including September 2019. To compare disuse‐related and RET‐related transcriptional responses in the context of age, our search implemented the following inclusion criteria: (i) recruitment of healthy (i.e. uninjured and non‐diseased) volunteers aged either 18–35 years (young) and/or ≥60 years (older); (ii) employment of a disuse intervention (unilateral limb immobilization or bed rest) lasting ≥7 days and/or a RET protocol lasting ≥3 weeks; (iii) a within‐person study design in which rested (i.e. not acutely exercised) *m. vastus lateralis* samples were obtained both pre‐intervention and post‐intervention; and (iv) expression profiling undertaken using a non‐customized microarray (refer to original publications for RNA handling procedures). We chose to include only microarray data sets herein to limit the potential for technological bias(es) upon aggregating microarray and RNA‐sequencing data (particularly in the context of meta‐network analyses)^33^, ^34^ and because there was an insufficient number of RNA‐sequencing data sets available to conduct meta‐analyses on at the time of searching (one RET and one disuse).^25^, ^35^ In order to establish universal gene signatures of RET/disuse muscle adaptation, we did not exclude studies based on the type of disuse intervention (i.e. unilateral limb immobilization and bed rest) or RET protocol (i.e. intensity, frequency) employed. Our implemented minimum cut‐offs for the duration of disuse/RET intervention were chosen because disuse‐induced muscle atrophy is reproducibly detectable at ≥7 days,^12^, ^14^ while RET‐induced hypertrophic gains (and not swelling‐associated muscle hypertrophy)^36^ are detectable after 3 weeks in both young and older individuals.^37^, ^38^ In total, 11 data sets (3 × disuse^21^, ^39^, ^40^; 3 × young RET^20^, ^23^, ^24^; 5 × older RET^23^, ^24^, ^41^, ^42^, ^43^) across nine distinct studies were included in downstream analyses (*Table* 1).

**Table 1:** Summary of RET and disuse transcriptomic studies included in the meta‐analysis

Reference	Array accession	Intervention	Intervention details	Number of pre‐intervention/post‐intervention sample pairs	Age	Sex	Platform
Damas *et al*., 2018^20^	GSE106865	RET	10 weeks, lower‐body RET, three times per week	10 Y	26 ± 2 years (mean ± SD)	10 M	Illumina HumanHT‐12 V4.0 Expression BeadChip
Hangelbroek *et al*., 2016^41^	GSE117525	RET	24 weeks, progressive whole‐body RET, three times per week	41 O	70 ± 5 years (mean ± SD)	26 M 15 F	Affymetrix Human Gene 1.1 ST Array
Melov *et al*., 2007^42^	GSE8479	RET	26 weeks, progressive whole‐body RET, two times per week	14 O	65–79 years	6 M8 F	Illumina Sentrix HumanRef‐8 Expression BeadChip
Phillips *et al*., 2013^23^	GSE47881 (A) GSE47881 (B)	RET	20 weeks, progressive whole‐body RET, three times per week	9 Y 16 O	<35 years ≥64 years	6 M, 3 F 9 M, 7 F	Affymetrix Human Genome U133 Plus 2.0 Array
Raue *et al*., 2012^24^	GSE28422 (A) GSE28422 (B)	RET	12 weeks, progressive lower‐body RET, three times per week	14 Y 12 O	20–30 years 84 ± 3 years (mean ± SD)	8 M, 6 F 6 M, 6 F	Affymetrix Human Genome U133 Plus 2.0 Array
Tarnopolsky *et al*., 2007^43^	MEXP740	RET	12 weeks, unilateral leg progressive RET, three times per week	8 O	71 ± 2 (mean ± SEM)	8 M	Affymetrix Human Genome U95A
Abadi *et al*., 2009^39^	GSE14901	Disuse	14 days, ULI	24 Y	21 ± 2 years 21 ± 3 years (mean ± SD)	12 M 12 F	Affymetrix Human Genome U133 Plus 2.0 Array
Alibegovic *et al*., 2010^40^	GSE24215	Disuse	9 days, bed rest	10 Y	24–27 years	10 M	Agilent‐014850 Whole Human Genome Microarray 4x44K G4112F
Rullman *et al*., 2018^21^	GSE104999	Disuse	21 days, bed rest	12 Y	~27 ± 6 years (mean ± SD)	12 M	Affymetrix Human Gene 2.1 ST Array

F, female; GSE, array accession from the Gene Expression Omnibus; M, male; MEXP, array accession from ArrayExpress; O, older; RET, resistance exercise training; ULI, unilateral limb immobilization; Y, young.

## Data pre‐processing

Arrays were processed on a data set‐by‐data set basis in line with procedures as standard for their corresponding platform, using the limma and oligo R packages where appropriate.^44^, ^45^ Specifically, all data sets generated using an Illumina array platform were normalized using the ‘neqc’ algorithm,^46^ in which background correction was performed using negative control probes and between‐array quantile normalization performed using both negative and positive controls, with values consequently represented on the log2 scale. For data sets generated using an Agilent array platform, arrays were background corrected using the ‘normexp’ method^46^ and normalized across one another using quantile normalization, before values were transformed to be on the log2 scale. All data sets generated using an Affymetrix array platform were normalized using the Robust Multichip Average algorithm,^47^ of which comprised background correction via subtraction, quantile normalization, and probe‐level summarization via median‐polishing—with the net result being intensity values on the log2 scale. This was with the exception of the data set GSE14901, which was generated using the Affymetrix Human Genome U133 Plus 2.0 array platform (*Table* 1) but only available as MAS5‐calculated signal intensities. In which case, arrays were transformed to be represented on the log2 scale. For each data set, control probes and probes without a corresponding Entrez Gene ID were consequently removed and the expression of probes corresponding to the same Entrez Gene ID then averaged. Finally, data sets were filtered for the intersection of each of their remaining Entrez Gene ID's, with the net result being a consistent set of 8244 genes present in all data sets for use in downstream analyses.

### Gene‐level meta‐analysis of global expression changes

Differential expression pre‐intervention vs. post‐intervention was first estimated per gene within each separate data set using empirical Bayes‐moderated paired *t*‐tests, as implemented in the limma package for R.^45^ For each gene, individual (right‐tailed) *P* values were then aggregated using the Stouffer's method^48^ to obtain a single meta‐analysis *P* value of its differential expression for each of the following data set combinations: (i) the three disuse data sets; (ii) the three young RET data sets, and (iii) the five older RET data sets. In all cases, meta *P* values were corrected using the Benjamini–Hochberg method to control for false discovery rate (FDR) and genes defined as significantly differentially expressed (DE) if they met *all* of the following criteria: (i) a corrected meta *P* value ≤ 0.1, (ii) an absolute meta (mean) log fold‐change > 0.1, and (iii) a common direction of gene log fold‐change across all pertinent data sets. This strict overall criterion for differential expression was applied to ensure the robust detection of concordant gene expression changes across individual data sets within each condition.

### Network‐level meta‐analysis to identify universal gene patterns

To gain a more holistic understanding of universal gene regulation in each condition, we complemented the gene‐level meta‐analysis with network‐level meta‐analysis. In particular, we employed a ‘consensus’ network approach using the weighted gene co‐expression network analysis package implemented in R.^49^ Consensus networks comprise distinct gene clusters (‘modules’) that are commonly present in multiple independent data sets.^31^ Modules that compose a consensus network therefore represent reproducible co‐expression relationships in a given scenario that are reflective of the underlying biology rather than technical artefacts.^31^ Consensus networks were constructed for each of the following data set combinations: (i) the three disuse data sets, (ii) the three young RET data sets, and (iii) the five older RET data sets.

Initially, a signed weighted adjacency matrix (*Adj*) quantifying the connection strength between each pair of genes was derived for each data set as *Adj* = |0.5 × (1 + *Corr*)|^*ß*^, where *Corr* is the matrix of Pearson's correlation coefficients that indicate the degree of similarity in expression pattern between any two given genes of that data set. The exponent *ß* was chosen per data set in accordance with the scale‐free topology criterion^50^ as the lowest integer for which the corresponding scale‐free topology fitting index metric achieved an appropriately high value (≥0.8). Each adjacency matrix was then converted into a topological overlap matrix (TOM), in which each entry provides a measure of the relative interconnectedness (‘common connections’) between a given pair of genes within a given data set. For each data set combination, TOMs were made comparable via calibration by single quantile scaling, with a consensus TOM (cTOM) then defined in each case by taking the component‐wise (‘parallel’) minimum of the associated calibrated TOMs.

Each cTOM was then converted into a consensus dissimilarity measure (dis‐cTOM = 1 − cTOM), with consensus networks consequently built for each data set combination via hierarchical clustering of their respective dis‐cTOMs using average linkage as a distance metric. The modules of each consensus network were subsequently determined using the *cutreeDynamic* algorithm,^51^ with a minimum module size of 50 genes selected so as to obtain moderately large and distinct modules in each instance, minimizing potential transcriptional noise that can occur when detecting gene modules in smaller‐sized data sets.^52^, ^53^ Finally, the composite expression of genes within a given consensus module was calculated on a per data set basis by taking the first principal component of module gene expression: herein referred to as the module ‘eigengene’. Each data set therefore has an eigengene per module of its associated consensus network.^32^ Modules within each consensus network were consequently merged if they were highly correlated (minimum eigengene correlation across its data sets > 0.75).

After constructing each consensus network, network‐level meta‐analysis was undertaken in similar fashion to above, but with the focus instead being on establishing differentially regulated consensus modules within each network. As such, differential regulation pre‐intervention vs. post‐intervention was first estimated per module eigengene of each individual data set. Then, the individual (right‐tailed) *P* values of differential eigengene expression in each data set were aggregated per consensus module to calculate a single, corrected meta‐analysis *P* value of that module's differential regulation. In any case, a consensus module was defined as being significantly differentially regulated using the same criteria as outlined above, but with the requisite for criterion (iii) instead being a common direction of *eigengene* change across all pertinent data sets, rather than a common direction of gene log fold‐change.

### Establishing concordant and discordant gene patterns across conditions

To determine common and uniquely regulated genes across conditions of interest, we utilized the rank–rank hypergeometric overlap (RRHO) algorithm,^54^ in which genes were ranked on sign of meta log fold‐change multiplied by the negative log10 of their corrected meta *P* value. Specific comparisons made were as follows: (i) young RET vs. disuse and (ii) young RET vs. older RET. In each case, commonly regulated genes were defined as those significantly DE in both conditions *and* present within the optimal overlapping gene set(s) between conditions. Uniquely regulated genes in each case were then defined as those significantly DE in a single condition *and not* present within the optimal overlapping gene set(s) between conditions. When comparing between young RET and disuse, commonly regulated genes were further defined on the basis of concordant vs. divergent regulation.

Inferring common and unique regulation at the network‐level is slightly more intricate than is at the individual gene‐level. Indeed, because network construction and module detection are *unsupervised* processes, individual networks are highly unlikely to be direct mirror images of one another in terms of their precise module compositions. Even then, module labels are arbitrary, and so, it would not necessarily hold true that (e.g.) module ‘M1’ in one network would equal module ‘M1’ of another network. There is also the potential that a particular set of genes form a module in one network but not another due to unique underlying biology in a given condition. To therefore make individual consensus networks more aligned to one another for comparison, we consequently utilized the weighted gene co‐expression network analysis *matchLabels* function.^55^ With this function, module gene compositions are compared between a ‘reference’ and ‘source’ network using Fisher's exact test. Modules in the source network are then subsequently renamed in line with the module labels of the reference network so that modules between networks with a significant number of overlapping genes also then have the same label (but such that no two modules of the source network are renamed with the same label of the reference network). Thus, modules with the same label in both networks have a significant number of overlapping genes, but not necessarily identical gene compositions, and modules with labels unique to a single network are not recapitulated in the other network.^55^ Clearly, this can be done only for two networks at any one time. Because our aim was to compare the young RET consensus network with both the disuse and older RET consensus networks, the young RET consensus network was thus used as the reference network, and modules of the disuse and older RET consensus networks were relabelled to be in line with those comprising the young RET consensus network. Note, it does not necessarily then hold true that the disuse and older RET consensus networks are comparable with one another. However, comparing these conditions was not the aim of this work. After this module calibration process, we were then able to make inferences on (un)common and unique module regulation by comparing across the differentially regulated consensus modules of: (i) the young RET and disuse networks and (ii) the young RET and older RET networks.

### Functional annotation of (un)loading‐associated gene patterns

The functional characteristics of DE genes and differentially regulated consensus network modules were derived by testing their comprising gene lists for both Gene Ontology enrichment and pathway enrichment, using the Enrichr web server.^56^ In the case of Gene Ontology analysis, we focused on enrichment for Biological Process terms.^57^ For pathway analysis, we focused on enrichment for terms contained within the Reactome Pathway Database.^58^ In all instances, terms with a Benjamini–Hochberg corrected *P* value < 0.05 were defined as being enriched.

### Network‐driven identification of hub genes as candidate mechanistic targets

In order to identify candidate regulatory molecules of muscle (mal)adaptation to RET and/or disuse, we first deduced consensus hub genes contained within our differentially regulated consensus network modules.^32^, ^59^ In line with the overarching concept of consensus network analysis, consensus hub genes represent module hub genes that are common in multiple independent data sets, the identification of which can be more useful than a gene‐level meta‐analysis *P* value for identifying biologically meaningful gene lists.^32^ Here, hub genes in consensus modules were derived using the consensus module membership metric—a measure that is strongly related to the ‘intramodular connectivity’ metric (another common measure traditionally used for the purposes of hub gene feature selection), but with the added benefit of being more suitable for candidate gene screening during network‐based meta‐analyses.^32^, ^60^ In brief, a module membership value for each gene was first calculated on a per data set basis as the correlation between its individual gene expression profile in that data set and the data set‐specific module eigengene (with associated *P* values being one‐sided to account for the fact that signed networks were constructed). Then, a consensus module membership value for each gene was derived as the *Z*‐score obtained from aggregating its gene–eigengene correlation across each pertinent data set using Stouffer's method.^32^ Consensus module genes with a consensus module membership value above the 85th percentile were then defined as consensus hub genes. Finally, we overlaid gene‐level and network‐level feature selections, which in the context of transcriptomic meta‐analysis may be a particularly useful approach to take when attempting to prioritize for the most biologically relevant candidate targets, because resultant genes are inherently characterized by robust regulation *and* interlinkage.^32^ Visualizations were generated using Cytoscape (v3.7.1).^61^

## Results

We first applied gene‐level meta‐analysis to identify robust gene expression changes. The total numbers of DE genes identified for each condition (disuse and younger/older RET) are shown in *Table* 2, with full lists of DE (plus RRHO) genes provided in *Table* S1. Overall, the number of DE genes observed post‐RET was considerably higher in young vs. older muscle (*Table* 2). To gain a more holistic understanding of molecular networks associated with atrophy/hypertrophy, we performed consensus network construction of gene co‐regulation, summarized in *Table* 3 (gene‐module assignments shown in *Table* S2). We next performed meta‐analysis of each module's eigengene (*Methods*). The number of differentially regulated consensus modules under each condition were 14 during muscle disuse (7 up‐regulated and 7 down‐regulated), 9 in young muscle following RET (7 up‐regulated and 2 down‐regulated), and 1 (down‐regulated) in older muscle following RET (*Table* S2). Enriched functional terms for DE (plus RRHO) gene lists and differentially regulated consensus modules are given in *Tables* S3 and S4, respectively.

**Table 2:** Total numbers of universally differentially expressed genes following RET in young and older muscle, as well as following disuse in young muscle

Variable	RET		Disuse
	Young	Older	Young
Up‐regulated	613	219	898
Down‐regulated	257	207	932

RET, resistance exercise training.

**Table 3:** Module characteristics of the consensus networks constructed for each of the young RET, older RET, and muscle disuse data set combinations

Characteristic	Consensus network		
	Young RET	Older RET	Disuse
Total modules	31	34	25
No. genes per module			
Mean	266	242	330
Range	72–667	84–734	79–992

RET, resistance exercise training.

### The transcriptional response to disuse is not simply the inverse of the transcriptional response to resistance exercise training

**Figure 1:**
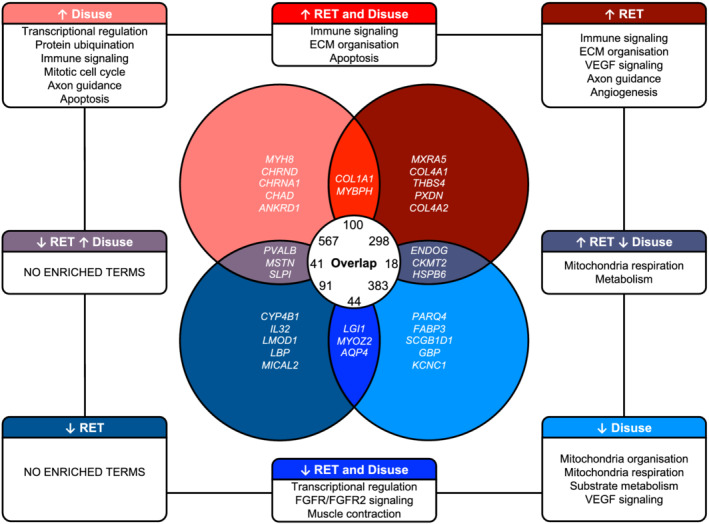
Comparison of gene‐level expression changes with RET vs. disuse. Venn diagram illustrates the degree of overlap between RET‐induced and disuse‐induced transcriptomic changes for all possible permutations, as determined via rank–rank hypergeometric overlap (RRHO) analysis. Outer boxes provide representative summaries of enriched Gene Ontology/Reactome Pathway terms for genes pertaining to each given scenario. The top 5 common and uniquely regulated genes when ranked by meta log2 fold‐change (as based on RRHO results) are provided within associated segment of the Venn diagram. Note: for concordant and discordant RRHO gene lists, only the intersection of top 5 ranking genes across each associated scenario is provided. ECM, extracellular matrix; RET, resistance exercise training.

At the individual gene‐level, we observed that (i) most gene changes are unique to disuse or RET in isolation and (ii) genes that do overlap are predominantly DE in the same direction, while a limited number of overlapping genes display inverse expression patterns between disuse and RET (*Figure* 1, *Table* S3). Disuse was uniquely characterized by up‐regulation of genes involved in protein ubiquination and mitotic cell cycle, while RET alone was characterized by up‐regulation of angiogenesis‐related genes (*Figure* 1, *Table* S3). Disuse and RET both resulted in up‐regulation of genes involved in immune signalling, some were the same genes while some were unique to each condition. Similarly, certain genes involved in ECM organization were up‐regulated by both disuse and RET, whereas others were only up‐regulated by RET (*Figure* 1, *Table* S3). Thus, while divergent muscle growth responses to disuse vs. RET might be partially underpinned by distinct immune system/ECM remodelling molecular responses, some pleiotropic regulation might exist within commonly regulated aspects of these pathways. Lastly, disuse and RET induced inverted expression profiles for 59 genes (*Figure* 1, *Table* S3). Eighteen were genes down‐regulated by disuse but up‐regulated by RET, which were heavily enriched for mitochondrial respiration processes (*Figure* 1, *Table* S3). A further 41 genes were up‐regulated by disuse but down‐regulated by RET, but these failed to cluster to any functional terms (*Figure* 1, *Table* S3). Interestingly, among the top five ranked DE genes in both conditions (i.e. top 5 down‐regulated by RET *and* top 5 up‐regulated by disuse of inverted responses) was myostatin (*MSTN*), a key molecule involved in the regulation of skeletal muscle mass^62^, ^63^ (*Figure* 1).

**Figure 2:**
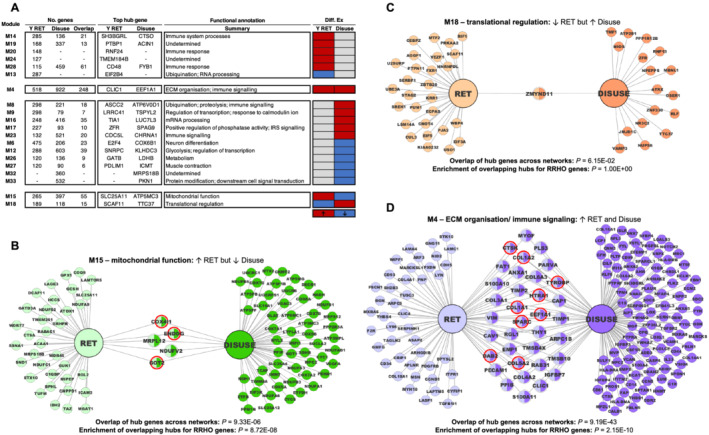
Consensus network analysis of RET vs. disuse responses in young muscle. Panel (A): Consensus modules shown are those differentially regulated by RET and/or disuse in young muscle. Red and blue shading denote significant up‐regulation and down‐regulation following each condition (vs. baseline), respectively. In all cases, functional summaries are representative of enriched Gene Ontology/Reactome Pathway terms for each given module. Also provided is the top ranked hub gene for each module per consensus network. Panels (B)–(D): Hub gene network visualizations for consensus modules concordantly or inversely regulated by RET and disuse in young muscle. Each visualization illustrates all corresponding hub genes of the given consensus module on a per condition basis, with dual‐coloured nodes thus representing genes that are overlapping consensus module hubs for both given conditions. Red borders (if any) illustrate the overlapping hub genes in each case that also appear among the corresponding list of RRHO genes for that particular scenario (e.g. up‐regulated by RET but down‐regulated by disuse). RET, resistance exercise training; RRHO, rank–rank hypergeometric overlap.

**Figure 3:**
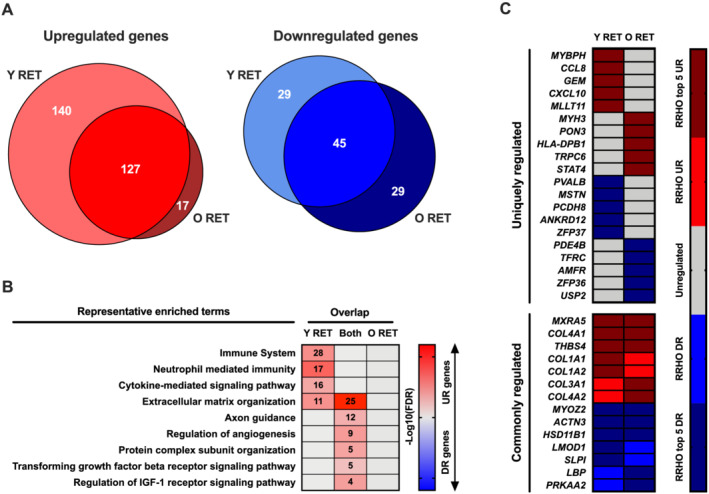
Comparison of gene‐level expression changes in young vs. older muscle following RET. Panel (A): Venn diagrams depicting the degree of overlap between up‐regulated and down‐regulated genes post‐RET in young vs. older muscle, as based on RRHO analyses. Panel (B): Representative enriched Gene Ontology/Reactome Pathway terms for common and uniquely regulated genes post‐RET in young vs. older muscle. A number of genes enriched in a given term are provided within associated boxes of the heatmap. Strength of colour shading depicts the magnitude of enrichment significance, given by the negative log10 of that term's enrichment FDR *P* value (with darker shading analogous with a stronger FDR *P* value). Panel (C): Top 5 common and uniquely regulated genes (up‐regulated and down‐regulated) per age group, ranked by meta log2 fold‐change (as based on RRHO results). Lighter shading denotes differential regulation for that age group, with darker shading indicating gene is among the top 5 ranked by meta log2 fold‐change for a given scenario (e.g. uniquely up‐regulated post‐RET in young vs. older muscle). RET, resistance exercise training.

Network‐level meta‐analysis revealed similar themes as the gene‐level approach. Both disuse and RET were characterized by separate up‐regulation of immune signalling networks, common up‐regulation of further immunity pathways and ECM organization networks, and inverse (down‐regulated by disuse and up‐regulated by RET) regulation of a mitochondrial function network (*Figure* 2A, *Table* S4). Additionally, network meta‐analysis revealed a divergent disuse vs. RET profile characterized by a disuse up‐regulated ‘translation‐related’ molecular network (M18) that was down‐regulated by RET (*Figure* 2A, *Table* S4). Next, we derived hub genes of differentially regulated consensus modules by screening for genes with robustly high intramodule membership, returning a base list of candidate regulatory molecules of muscle adaptation (708 and 319 hub genes in the young disuse and RET networks, respectively) (*Table* S5). For the three consensus modules with altered regulation in the disuse and RET networks (M4, M15, and M18), we further prioritized hub genes for those displaying a high intramodular hub status under both conditions. All three modules each had at least one gene with shared hub gene status across both respective networks, with the overlap reaching significance in two modules (M4 and M15) (*Figures* 2B–3D). Common hub genes identified in the divergent disuse vs. RET profile were as follows: *COX4I1* (Cytochrome c oxidase subunit 4 isoform 1), *ENDOG* (Endonuclease G), *MRPL12* (Mitochondrial Ribosomal Protein L12), *NDUFV2* (NADH:Ubiquinone Oxidoreductase Core Subunit V2) and *GOT2* (Glutamic‐Oxaloacetic Transaminase 2) for mitochondrial responses, and *ZMYND11* (Zinc Finger MYND‐Type Containing 11) for translational regulation (*Figures* 2B and 3C, *Table* S5). We further screened our base candidate gene lists for module hub genes contained within the corresponding RRHO gene list (e.g. for a module up‐regulated only in the RET network vs. disuse network, we considered the RRHO gene list uniquely up‐regulated by RET vs. disuse, etc.) (*Table* S5). For consensus modules across the disuse and RET networks, *COX4I1*, *ENDOG*, and *GOT2* were highlighted among inverted mitochondrial responses (*Figure* 2B), and *COL1A1* (Collagen type 1 alpha 1), *COL1A2* (Collagen type 1 alpha 1), *COL5A2* (Collagen type 5 alpha 2), *CTSK* (Cathepsin K), *DAB2* (Disabled homologue 2), *EEF1A1* (Eukaryotic translation elongation factor 1 alpha 1), *HTRA1* (HtrA Serine Peptidase 1), *SPARC* (Secreted protein acidic and rich in cysteine), *TYROBP* (TYRO protein tyrosine kinase binding protein) highlighted among the commonly regulated ECM/immune responses after disuse and RET (*Figure* 2D, *Table* S5).

### Older muscle displays a blunted transcriptional response to resistance exercise training compared with younger muscle

Virtually all genes up‐regulated by RET in older muscle were also up‐regulated in young muscle, but a substantial number of genes (*n* = 140) up‐regulated post‐RET in younger muscle were not similarly up‐regulated in older muscle (*Figure* 3A, *Table* S1). Genes up‐regulated by RET in young and older muscle were primarily involved in ECM organization, axon guidance, and the regulation of angiogenesis (*Figure* 3B, *Table* S3). The 140 genes uniquely up‐regulated post‐RET in younger muscle clustered to additional ECM organization processes, as well as immune‐related signalling pathways (*Figure* 3B, *Table* S3). Interestingly, 11 of the 140 uniquely up‐regulated young RET genes aligned to ECM organization, some of which have established roles in mechanical force transmission [integrin subunits *ITGAM* (Integrin Subunit Alpha M), *ITGAE* (Integrin Alpha E), and *ITGB2* (Integrin Subunit Beta 2)], regenerative pathways [*TNC* (Tenascin C)], and collagen reinforcing processes [*PLOD2* (Procollagen‐lysine, 2‐oxogluterate 5‐dioxygenase 2)] (*Figure* 3B, *Table* S3). Additionally, 28 of the 140 genes uniquely up‐regulated in young RET muscle aligned to the immune system, including several caspase proteolytic enzymes [*CASP1* (Caspase 1) and *CASP3* (Caspase 1)]. Within the top 5 ranked genes uniquely up‐regulated in young RET muscle, the highest‐ranked gene was the myosin binding protein *MYBPH* and also included two inflammation/immune‐related transcripts [*CCL8* (Chemokine ligand 8) and *CXCL10* (C‐X‐C Motif Chemokine Ligand 10)] (*Figure* 3C). Despite the clear young muscle‐specific ECM signature, several collagens were also commonly regulated in both young and older muscle following RET [*COL1A1* (Collagen Type I Alpha 1 Chain), *COL1A2* (Collagen Type I Alpha 2 Chain), *COL3A1* (Collagen Type III Alpha 1 Chain), and *COL4A2* (Collagen Type IV Alpha 1 Chain)] (*Figure* 3C). Substantially, fewer genes were down‐regulated by RET in both younger and older muscle (*Figure* 3A, *Table* S1), all of which failed to cluster to any functional terms (*Figure* 3B, *Table* S3).

**Figure 4:**
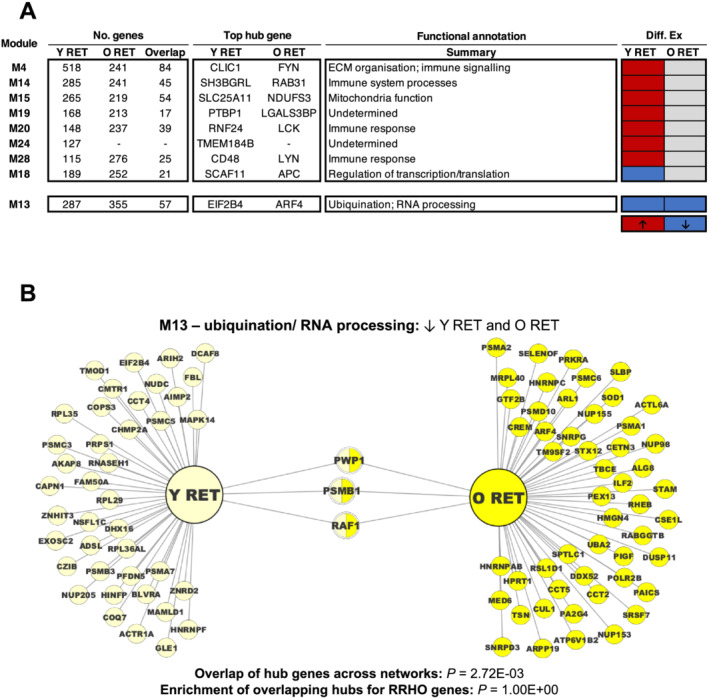
Consensus network analysis of RET responses in young muscle vs. older muscle. Panel (A): Consensus modules shown are those differentially regulated by RET in young muscle and/or older muscle. Red and blue shading denote significant up‐regulation and down‐regulation following RET (vs. baseline), respectively. In all cases, functional summaries are representative of enriched Gene Ontology/Reactome Pathway terms for each given module. Also provided is the top ranked hub gene for each module per consensus network. Panel (B): Hub gene network visualization for M13; the consensus modules concordantly down‐regulated by RET in young and older muscle. Visualization illustrates all corresponding hub genes of the given consensus module on a per age basis, with dual‐coloured nodes thus representing genes that are overlapping consensus module hubs for both ages. Red borders (if any) illustrate the overlapping hub genes in each case that also appear among the corresponding list of RRHO genes for that particular scenario (i.e. commonly down‐regulated by RET in both young and older muscle). RET, resistance exercise training.

At the network‐level, similar to gene‐level analysis, only young muscle displayed a post‐RET up‐regulation of genes comprising several immune signalling molecular networks and ECM organizational response (*Figure* 4A, *Table* S4). However, network analysis provided functional insight into down‐regulated RET genes, with both younger and older RET being characterized by the down‐regulation of similar ‘ubiquination/RNA processing’‐related molecular networks (M13) (*Figure* 4A, *Table* S4), the common hub genes of which were identified to be *PWP1* (Periodic Typtophan Protein 1), *PSMB1* (Proteasome 20S Subunit Beta 1) and *RAF1* (c‐RAF) (*Figure* 4B, *Table* S5). Overlaying corresponding consensus module hub gene and RRHO gene lists further identified *ARF3* (ADP‐ribosylation factor 3), *ARPC1B* (Actin Related Protein 2/3 Complex Subunit 1B), *BPHL* (Biphenyl Hydrolase Like), *CAP1* (Cyclase Associated Actin Cytoskeleton Regulatory Protein 1), *EMP1* (Epithelial Membrane Protein 1), *HIF1A* (Hypoxia‐Inducible Factor 1‐alpha), *LST1* (Leukocyte Specific Transcript 1), *RAB31* (Ras‐related protein Rab31), *SERPINI1* (Serpin Family I Member 1), *TUFM* (Tu Translation Elongation Factor), and *VIM* (Vimentin) as prime candidates of RET‐induced muscle remodelling specifically in younger age (*Table* S5).

## Discussion

Herein, we integrated gene‐level and network‐level approaches into a single transcriptomic meta‐analysis pipeline to identify robust biosignatures and candidate regulatory molecules of muscle adaptation under societally important atrophic and hypertrophic conditions. Our findings reveal inverted expression profiles after disuse compared with RET and a reduced transcriptional responsiveness of older muscle to RET. This analysis should serve as a solid foundation from which to understand and develop targeted interventions against disuse‐related atrophy and age‐related responsiveness to RET, with potential relevance to other diseases characterized by muscle loss (e.g. muscular dystrophy, cancer, and rheumatoid arthritis).

### Despite largely distinct transcriptional signatures, mitochondrial and translational regulation display converse responses to disuse and resistance exercise training

Disuse and RET elicit opposing muscle phenotypes (i.e. atrophy and hypertrophy, respectively),^37^, ^64^ thus one might anticipate opposing transcriptional responses. Consistent with this notion, we illustrate that declines in a subset of mitochondrial genes and increases in some translational regulation genes in response to disuse occur in a directly opposing manner following RET. However, we also find that the majority of gene changes induced by disuse and RET are entirely distinct from one another, implying muscle adaptations to unloading vs. loading are largely separate molecular processes. Thus, a key finding from our meta‐analysis is that the mechanisms of disuse are not simply the converse of RET and cannot be derived from studying muscle loading alone. These observations of RET failing to target all the molecular changes associated with disuse, might reflect reports that RET is incompletely effective at countering disuse atrophy^18^ and provides a robust molecular platform from which to determine the precise disuse‐RET relationship.

Mitochondria are critical organelles regulating muscle metabolism, health, and function.^65^ Indeed, mitochondrial dynamics and/or respiratory capacity decrease during disuse^66^ and increase during RET.^67^ Our findings support and extend recent meta‐analyses reporting down‐regulated mitochondrial profiles in disuse alone,^28^ by establishing an opposing mitochondrial profile between disuse and RET and establishing a putative molecular basis for divergent mitochondrial adaptations. We identified *COX4I1* as one of the top genes in the divergent mitochondrial response to disuse and RET. *COX4I1* is an isoform of electron transport chain complex IV, which is often used as a common marker of mitochondrial content^68^ and mitochondrial oxidative metabolism.^69^ Given that complex IV is a terminal electron acceptor for the proton motive force driving ATP production,^70^ it is plausible that *COX4I1* mechanistically contributes to enhanced mitochondrial respiratory capacity following RET.^67^ *GOT2*, which acts within the malate–aspartate shuttle wherein NADH electrons are delivered to electron transport chain complex I,^71^, ^72^, ^73^ was also identified as displaying a divergent response to disuse and RET. While a paucity of data exists in healthy humans, in type II diabetics (typically characterized by impaired mitochondrial function),^74^, ^75^ *GOT2* expression is lower compared with control patients, which is also increased after endurance exercise training.^73^ These observations suggest that: (i) prominent opposing transcriptional profiles could partially explain impaired (disuse) vs. improved (RET) mitochondrial respiratory function and/or dynamics; and (ii) interventions targeting mitochondria, such as electron donors, might reproduce some of the beneficial health effects of RET during disuse.

Genes involved in translational regulation were also identified as up‐regulated by disuse and down‐regulated by RET. While seemingly paradoxical given that translation is a key process driving MPS^76^ and, therefore, hypertrophy,^77^, ^78^ RET‐induced decreases in translational regulation might reflect increased translational efficiency, as previously shown.^25^, ^79^ Such improved translational efficiency would reduce dependency on up‐regulation of proteins involved in the translation machinery to optimize anabolism. Corroborating this theory, a recent meta‐analysis (which had a significantly broader inclusion criteria and focused on differential gene analysis alone) also identified an inverted ‘mRNA metabolism’ signature between disuse and RET.^28^ Thus, alternate regulation of translational machinery presents as a robust molecular feature of disuse vs. RET. We also identified *MSTN* as one of the top genes displaying an increase in response to disuse and a decrease in response to RET, supporting previous findings.^28^ Myostatin is one of the best‐described regulators of muscle mass, playing a key role in muscle atrophy by synergistically impairing anabolic Akt signalling while activating the catabolic ubiquitin proteasome pathway to promote a negative net protein balance.^62^ Targeted analyses have previously reported lowered myostatin expression after RET and increased expression post‐immobilization in young, healthy volunteers.^80^, ^81^ These observations suggest that interventions targeting translational regulation might also be efficacious during disuse. Notably, inhibition of myostatin has very recently been shown to ameliorate muscle loss in rodents subject to disuse in spaceflight,^82^ suggesting further clinical trials are warranted, as previously suggested.^62^, ^63^

### Blunted transcriptional responses to resistance exercise training might influence age‐related anabolic resistance

Physiologically, RET can induce muscle hypertrophy in youth^37^ and in older age.^83^ However, muscle growth responses to RET are suboptimal in older age (termed ‘anabolic resistance’ to RET),^79^, ^84^ albeit through incompletely defined mechanisms. In line with age‐related responses to loading in rodents,^85^ we found the young RET response to be characterized by a much more substantial transcriptional response (i.e. 140 uniquely up‐regulated genes in young vs. only 17 in older muscle), that encapsulates most of the older RET profile. Thus, while insufficient data on protein metabolism and/or muscle mass within our analyses precludes functional associations between transcriptional changes and true anabolic resistance (i.e. blunted RET‐induced muscle protein synthesis/growth responses), our findings suggest that anabolic resistance to RET might be partly underpinned by a concomitant age‐related insensitivity to RET at the transcriptional level.

Remodelling of the ECM plays a pivotal role in muscle maintenance^86^ and, accordingly, RET‐responsive genes commonly regulated in both age groups included ECM‐related collagens. Therefore, ageing muscle appears to retain capacity for RET‐induction of some individual ECM collagens, similarly to young people. However, muscular benefits of functional ECM are not limited to maintaining physical integrity via structural collagens. Indeed, mechanical forces exerted during exercise are transduced through the ECM into focal adhesion structures, for conversion to biochemical signals (‘mechanotransduction’^86^, ^87^). In this context, several genes uniquely up‐regulated in younger muscle that map to ECM‐related pathway terms also have established roles in mechanotransduction. These included integrin‐related genes (*ITGAM*, *ITGB2*, and *ITGAE*); ECM‐linked transmembrane components that mechanically mediate wide‐ranging cellular processes^88^, ^89^, ^90^ including muscle metabolic health, structural integrity, and growth.^91^ Other mechanotransduction associated genes showing this trend included *PLOD2*, a collagen crosslinking molecule providing tensile integrity and collagen organization,^92^ and *TNC*, that supports muscle matrix remodelling^93^ and displays abnormal muscle damage/repair responses in older muscle.^94^ Network analysis corroborated young RET muscle‐specific mechanotransduction signatures, including overlapping young‐specific hub/RRHO features *VIM* (involved in hypertrophic signalling downstream of the integrin cascade^95^) and *ARPC1B* (an Arp2/3 subunit required for normal integrin adhesome assembly^96^). Lastly, the top up‐regulated gene in young, but not older RET responses was the myosin binding protein, *MYBPH*. While relatively uncharacterized, *MYBPH* up‐regulation is a feature of severe myopathy,^97^, ^98^ thus the reasons for increased *MYBPH* in healthy muscle RET responses is unclear. However, *MYBPH* also functions in the integrin associated Rho kinase 2 > cofilin2 pathway,^97^, ^99^ further implicating abnormal mechanotransduction in ageing muscle RET responses. Combined, these findings suggest some older muscle collagens respond appropriately to RET, but faltering ECM‐connected transmembrane components might functionally manifest as disorganized ECM and impair mechanically mediated remodelling/growth pathways. Targeting this force transduction axis might, therefore, represent a promising avenue for targeted therapeutic strategies.

Another functional class of diminished age‐related transcriptional responses to RET was inflammatory pathways. The presence of chronic low‐grade inflammation is well‐established in the aetiology of ageing muscle decline,^100^ which would narrow the physiological inflammatory range and manifest as a loss of RET‐induced inflammation gene signatures, as observed herein. Interrogation of individual inflammatory‐responsive genes identified caspase activation as a central theme. Caspases stimulate essential proteolysis, removing non‐functional myofibrillar proteins to facilitate deposition of new proteins for hypertrophic adaptation,^101^ such that impaired caspase transcript activation in ageing could hinder effective sarcomere remodelling during RET. Moreover, two of the top up‐regulated genes in young RET muscle were inflammatory chemokine transcripts *CCL8* and *CXCL10*, the abnormal regulation of which associates with ageing muscle regenerative decline (*CCL8* ^102^) and impaired macrophage > satellite‐cell‐mediated myogenesis (*CXCL10* ^103^). Overall, a consistent pattern emerges where failure to mount proper RET‐induced inflammatory responses could hinder ageing muscle regenerative capacity and growth responses.^104^

Young muscle also exhibited consistent RET up‐regulation of genes involved in immune‐related signalling, which was absent in older muscle. It is well‐established that the immune system plays an integral role in RET‐induced muscle hypertrophy, in part through mediating satellite‐cell dependent muscle repair and regeneration.^105^ These findings thus provide further support to the transcriptional theme that poorer RET adaptation of older muscle may be underpinned, at least in part, by age‐related impairments in muscle regenerative capacity.^106^ Across the three immune‐specific network modules uniquely up‐regulated post‐RET in young muscle (M14, M20, and M28), we identified three hub genes that were also robustly up‐regulated at the gene‐level exclusively in young muscle following RET, namely, *HIF1A*, *SERPINI1* (both M14), and *LST1* (M20). The identification of the oxygen‐sensitive subunit of the HIF‐1 transcription factor, *HIF1A*, is notable for its expression in nearly all innate and adaptive immune populations^107^ and its expression in most tissues including skeletal muscle.^108^ HIF‐1 regulates the transcription of >100 genes, many of which promote angiogenesis to increase nutrient and oxygen transport capacity to muscle. Signalling downstream of *HIF1A* is also proposed to facilitate muscle regeneration/growth by driving proliferation of satellite cells.^109^ As such, *HIF1A* could represent a promising candidate for understanding attenuated adaptations of older muscle to RET.

### Study limitations

Variability across study protocols is a common caveat of meta‐analyses and, despite strict inclusion/exclusion criteria, the duration of the disuse and RET programmes differed between studies within our analyses. Nonetheless, a critical criterion was capturing atrophic/hypertrophic phenotypes to which transcriptional profiles could be associated. Minimum disuse/RET duration cut‐offs were, therefore, implemented that were sufficient to obtain muscle mass changes, as opposed to precisely matching intervention durations. It is also unfortunate that detailed physiological data (e.g. individual changes in muscle mass, strength and/or metabolism) was not sufficiently available across our analysed data sets to assess for molecular markers that associate with the magnitude of interindividual variability in physiological responses to disuse and RET, as previously reported for the ageing exercise response.^23^, ^110^ Achieving this would promote identification of candidate molecules that account for the well‐established variance in adaptive responses to disuse and/or exercise training^14^, ^111^, ^112^ to identify the most promising therapeutic targets. Towards this end, future clinical studies would benefit from complementing health outcome measures with OMIC measurements to unlock the significant mechanistic potential of randomized controlled trials.

### Conclusions and future perspectives

In summary, we provide the first integrated gene‐level and network‐level meta‐analytical approach to elucidate robust molecular signatures and candidate drivers of muscle adaptation to disuse and RET in the context of age. Our findings show that limited gene features display inverted profiles to disuse vs. RET, but for the most part, transcriptional responses to disuse and RET are entirely distinct, suggesting muscle loading/unloading are largely independent molecular processes. We also identify diminished expression of ECM‐linked mechanotransduction and inflammatory pathways as key features of ageing muscle RET adaptations. By overlaying gene‐level *and* network‐level feature selections, we further identify candidate therapeutic targets characterized by robust regulation (gene‐level) and interlinkage (network‐level), which may aid the mitigation of, or accelerate recovery from, muscle disuse atrophy and potentially wider‐ranging atrophic diseases (e.g. *COX4I1*, *GOT2*, and *ENDOG*), and/or optimize RET‐induced hypertrophy across the lifespan (e.g., *HIF1A*).

## Funding

Colleen S. Deane acknowledges support from the Medical Research Council (MR/T026014/1). Craig R. G. Willis is supported by the Biotechnology and Biological Sciences Research Council‐funded South West Biosciences Doctoral Training Partnership (BB/J014400/1; BB/M009122/1). This work was partially supported by funding from BBSRC (grant BB/N015894/1 and BB/S002863/1). This research was also supported by the MRC Versus Arthritis Centre for Musculoskeletal Ageing Research (grant MR/P021220/1 and MR/R502364/1) and National Institute for Health Research Nottingham Biomedical Research Centre. The views expressed are those of the author(s) and not necessarily those of the NHS, the NIHR, or the Department of Health and Social Care.

## Conflicts of interest

Colleen S. Deane, Craig R. G. Willis, Bethan E. Phillips, Philip J. Atherton, Lorna W. Harries, Ryan M. Ames, Nathaniel J. Szewczyk, and Timothy Etheridge declare that they have no conflict of interest.

## Supporting information


**Table S1.** Supporting information

**Table S2.** Supporting information

**Table S3.** Supporting information

**Table S4.** Supporting information

**Table S5.** Supporting information
